# Oit3, a promising hallmark gene for targeting liver sinusoidal endothelial cells

**DOI:** 10.1038/s41392-023-01621-2

**Published:** 2023-09-12

**Authors:** Zhi-Wen Li, Bai Ruan, Pei-Jun Yang, Jing-Jing Liu, Ping Song, Juan-Li Duan, Lin Wang

**Affiliations:** 1 Department of Hepatobiliary Surgery, Xi-Jing Hospital, Fourth Military Medical University, 710032 Xi’an, China; 2 Center of Clinical Aerospace Medicine & Department of Aviation Medicine, Fourth Military Medical University, 710032 Xi’an, China

**Keywords:** Angiogenesis, Cell biology

## Abstract

Liver sinusoidal endothelial cells (LSECs) play a pivotal role in maintaining liver homeostasis and influencing the pathological processes of various liver diseases. However, neither LSEC-specific hallmark genes nor a LSEC promoter-driven Cre mouse line has been introduced before, which largely restricts the study of liver diseases with vascular disorders. To explore LSEC-specific hallmark genes, we compared the top 50 marker genes between liver endothelial cells (ECs) and liver capillary ECs and identified 18 overlapping genes. After excluding globally expressed genes and those with low expression percentages, we narrowed our focus to two final candidates: Oit3 and Dnase1l3. Through single-cell RNA sequencing (scRNA-seq) and analysis of the NCBI database, we confirmed the extrahepatic expression of Dnase1l3. The paired-cell sequencing data further demonstrated that Oit3 was predominantly expressed in the midlobular liver ECs. Subsequently, we constructed inducible Oit3-CreERT2 transgenic mice, which were further crossed with ROSA26-tdTomato mice. Microscopy validated that the established Oit3-CreERT2-tdTomato mice exhibited significant fluorescence in the liver rather than in other organs. The staining analysis confirmed the colocalization of tdTomato and EC markers. Ex-vivo experiments further confirmed that isolated tdTomato+ cells exhibited well-differentiated fenestrae and highly expressed EC markers, confirming their identity as LSECs. Overall, Oit3 is a promising hallmark gene for tracing LSECs. The establishment of Oit3-CreERT2-tdTomato mice provides a valuable model for studying the complexities of LSECs in liver diseases.

## Introduction

Liver sinusoidal endothelial cells (LSECs) represent a highly specialized population of endothelial cells (ECs) lining liver sinusoids.^1^ LSECs comprise a sizeable percentage (15-20%) of the overall hepatic cellular populace, rendering them the predominant non-parenchymal cell phenotype within the hepatic milieu.^2^ These cells play pivotal roles in modulating various pathophysiological processes within the liver, including liver regeneration, fibrosis, steatosis, and cancer.^2,3^ Their unique location and phenotype allow LSECs to regulate blood flow, nutrient exchange, immune responses, and the clearance of waste products in the liver microenvironment.^4^ Despite the recognized importance of LSECs, the identification of specific marker genes that uniquely characterize these cells remains a challenge.^5^ The absence of reliable markers hampers our ability to precisely characterize and isolate LSECs, limiting our understanding of their molecular profile and functional characteristics. Besides, the current unavailability of LSEC promoter-driven Cre mice, which are essential tools for investigations into cell-specific gene activities, has created a bottleneck in the research concerning LSEC-associated liver diseases.

LSECs hold a pivotal and indispensable role in maintaining the homeostasis of the liver endothelial niche,^6^ orchestrating hepatocytes,^7^ hepatic stellate cells (HSCs),^8^ and Kupffer cells (KCs)^9^ by enacting the intricate process of angiocrine signaling. Our research group has devoted considerable time and effort to investigating the regulatory function of LSECs in liver regeneration,^10^ fibrogenesis,^11,12^ and steatohepatitis.^13^ Previously, we and other researchers have predominantly relied upon traditional and canonical EC markers like CD31,^14^ VEGFR2,^15^ or VEGFR3^16,17^ to effectively label the hepatobiliary endothelium. However, none of them is liver-specific. Lyve1, which has been validated as a superior discriminator of LSECs, exhibits abundant expression in lymphatics and lung tissues.^18,19^ To precisely observe the impact of LSECs on liver pathology, we constructed Cdh5-CreERT transgenic mice.^11,20^ However, Cdh5 also known as VE-Cadherin, could be detected globally in murine endothelial cells.^19^ Hence, a compelling urgency arises in the quest for an exclusive marker gene that unequivocally delineates LSECs and the development of LSEC-driven Cre mice, which would serve as a catalyst for pioneering investigations into the intricate domain of the liver endothelial niche.

The advent of innovative methodologies such as single-cell sequencing and spatial transcriptomics has ushered in a new era of scientific inquiry,^21^ enabling unprecedented advancements in our comprehension of the intricate tapestry of genetic diversity and spatial organization within endothelial cells across various organs. For instance, Kaljcka J. et al. constructed a comprehensive global EC atlas of mice, providing a valuable resource for investigating the specific gene expression patterns of ECs derived from different organs.^19^ During single-cell sequencing, the spatial data related to specific cells is often lost due to tissue dissociation.^22^ The establishment of paired-cell sequencing, whereby mRNA from pairs of attached cells are sequenced and gene expression from one cell type is used to infer the pairs’ tissue coordinates,^23^ significantly addresses the gaps in our understanding of the precise localization of liver endothelial cells.^24^ By virtue of these advancing techniques, it will be easier for us to identify a subpopulation of ECs, which are strictly distributed in liver sinusoids.

Oncoprotein-induced transcript 3 (Oit3), also known as liver-specific zona pellucida domain-containing protein (LZP), has received limited scrutiny in prior investigations.^25,26^ OIT3 exhibits notable conservation in both humans and rodents, with initial findings delineating its primary expression at the hepatic locus, specifically within the confines of the nuclear membrane, and the secretion of a subset of OIT3 into the extracellular expanse.^27^ Earlier studies propose that OIT3 may play a role in hepatic development and differentiation. Additionally, few investigations focused on the role of Oit3 in liver metabolism and hepatocellular carcinoma development by its ability to promote M2-polarized macrophages and facilitate tumor cell invasion.^28–30^ However, previous research has not addressed the expression and function of OIT3 in endothelial cells, particularly in LSECs. In this manuscript, we employed a multi-single-cell RNA sequencing analysis to challenge the conventional markers previously employed to demarcate LSECs, thereby unraveling the potential link between Oit3 and LSECs. We provided accumulated data to prove that Oit3 is a LSEC-specific hallmark gene. Moreover, we constructed Oit3-driven CreERT2 transgenic mice and validated their effectiveness. Our research endeavors to offer reliable methodologies for investigating the intricate functionalities and mechanisms of LSECs in both physiological and pathological states.

## Results

### Single-cell RNA sequencing reveals liver endothelial marker genes

**Fig. 1:**
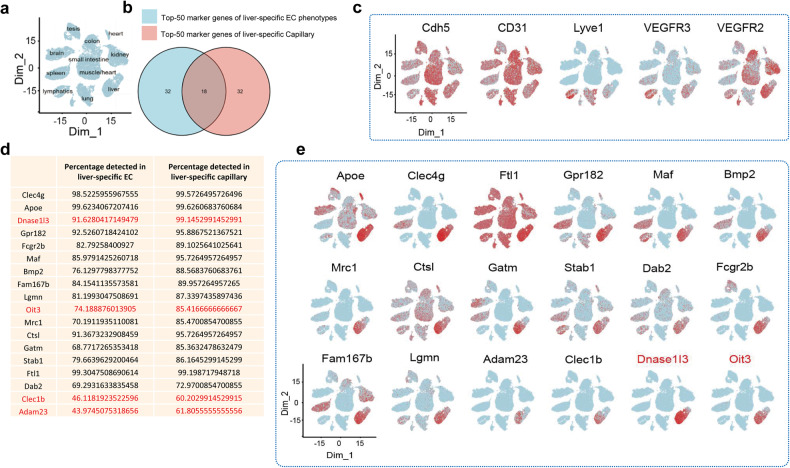
Single-cell RNA sequencing reveals liver endothelial marker genes. **a** t-SNE plot of endothelial cells in different organs. **b** Venn diagram of the top 50 marker genes of liver-specific EC phenotypes and the top 50 marker genes of liver-specific capillary. **c** t-SNE plot of traditional markers used to label LSECs, including Cdh5, CD31, Lyve1, Vegfr2 and Vegfr3. **d** The list of the names and the expressing percentage of the 18 overlapped genes. **e** t-SNE plot of the 18 genes (EC Atlas; https://carmelietlab.sites.vib.be/en/softwaretools/scCycle)

According to the transcriptome atlas of ECs established by Kaljcka et al. ^19^ the spatial expression of endothelial marker genes in different organs can be precisely exhibited. The clusters manifesting endothelial cells of different organs were shown in Fig. 1a. We then speculated the distribution of some well-known EC marker genes once used to LSECs,^1,31^ such as Cdh5, CD31, Lyve1, VEGFR3, and VEGFR2. However, none of them was liver-specific (Fig. 1c). Lyve1 and VEGFR2, which we used most to distinguish LSECs previously, expressed highly in the lung rather than the liver. Thus, exploring a better LSEC-specific marker gene seemed to be necessary.

Since LSECs are specialized liver capillary ECs,^32^ we then analyzed the top 50 marker genes of liver-specific EC phenotypes and the top 50 marker genes of the liver-specific capillary.^19^ On comparing these lists of genes, we found 18 that overlapped (Fig. 1b). The names and the expressing percentage of the 18 genes were listed in Fig. 1d. Moreover, the distribution of these 18 genes was observed in ECs of different organs relying on the single-cell RNA-sequencing (scRNA-seq) data. We found that only Adam23, Clec1b, Dnase1l3 (Deoxyribonuclease 1 Like 3), and Oit3 exhibited significant liver-specific expression (Fig. 1e). Given the relatively lower expression (<50%) of Adam23 and Clec1b in liver ECs (Fig. 1d), we selected Oit3 and Dnase1l3 as the two candidates for further evaluation.

### Oit3 outweighs Dnase1l3 in labeling LSECs

**Fig. 2:**
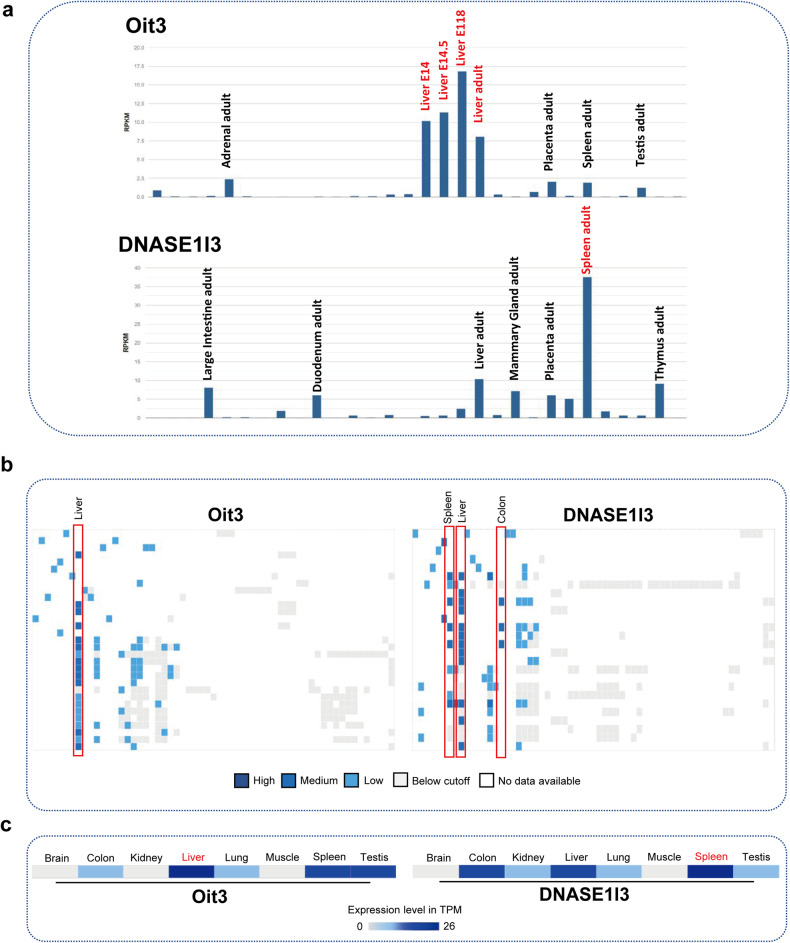
The global gene expression of Oit3 and Dnase1l3. **a** The relative expression of Oit3 and Dnase1l3 in distinct organs of embryos and adults in mice, data were collected from NCBI database. **b** The expression level of Oit3 and Dnase1l3 in distinct organs in mice with different strains (www.ebi.ac.uk/gxa/home). **c** The relative expression level of Oit3 and Dnase1l3 in distinct organs of C57 mice (www.ebi.ac.uk/gxa/home)

Concerning ECs, Oit3, and Dnase1l3 are expressed predominantly in the liver (Fig. 1e). However, whether they appeared in other kinds of cells of distinct organs was poorly understood. Therefore, we collected murine data from the NCBI database. To be noted, the high-level expression of Oit3 was observed in livers of embryo day 14 (E14), E14.5, E18, or adults, however, the largest expression of Dnase1l3 was detected in the spleen (Fig. 2a), indicating Dnase1l3 is not liver-specific. To confirm these findings, we further evaluated these two genes in the Expression Atlas (www.ebi.ac.uk/gxa/home). Consistently, high-level expression of Oit3 was found in liver again, but the enrichment of Dnase1l3 was investigated in spleen and colon of mice with different strains except for liver (Fig. 2b). Once we focused on the C57/BL6 mice, we found that the highest expression level of Oit3 and Dnase1l3 was inspected in liver and spleen respectively (Fig. 2c), which was consistent with the findings we observed through NCBI database.

**Fig. 3:**
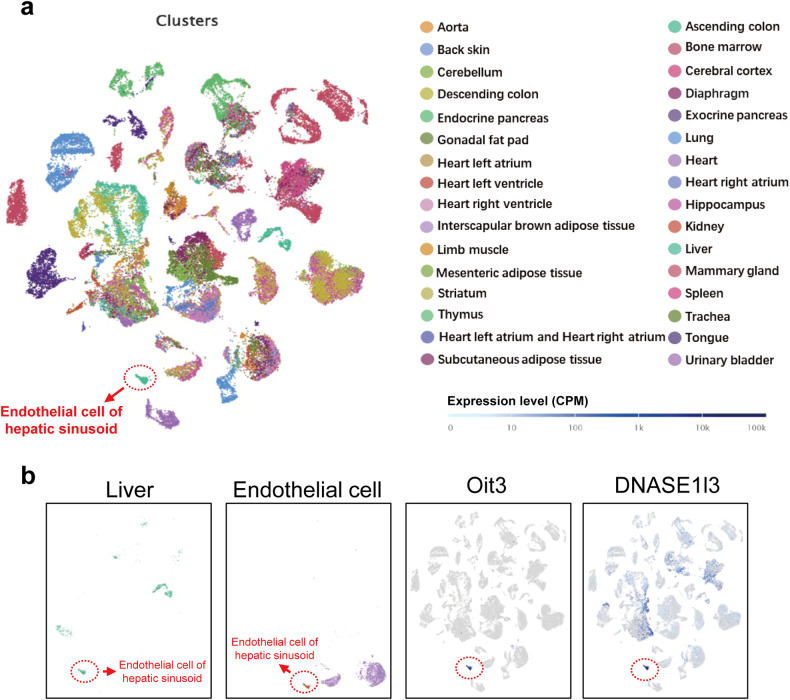
Single-cell RNA-seq of global cells. **a** The clusters of 20 distinct organs and tissues from the previously established Tabula Muris of scRNA-seq analysis of individual mice. **b** The t-SNE plot of cells in liver and all endothelial cells is shown in the left two panels, while the right two panels show the expression of Oit3 and Dnase1l3 across various organs and tissues (GSE109774)

Previously, a Tabula Muris of scRNA-seq analysis of 20 organs and tissues from individual mice was established.^33^ The clusters of distinct organs and tissues were demonstrated in Fig. 3. Interestingly, the spatial distribution of Oit3^+^ cells coincided entirely with hepatic sinusoid endothelial cells. However, the clusters representing Dnase1l3 exhibited in neither a liver- nor EC-specific manner. These data collectively confirmed that Oit3 should be the preferred gene to label LSECs in comparison with Dnase1l3.

**Fig. 4:**
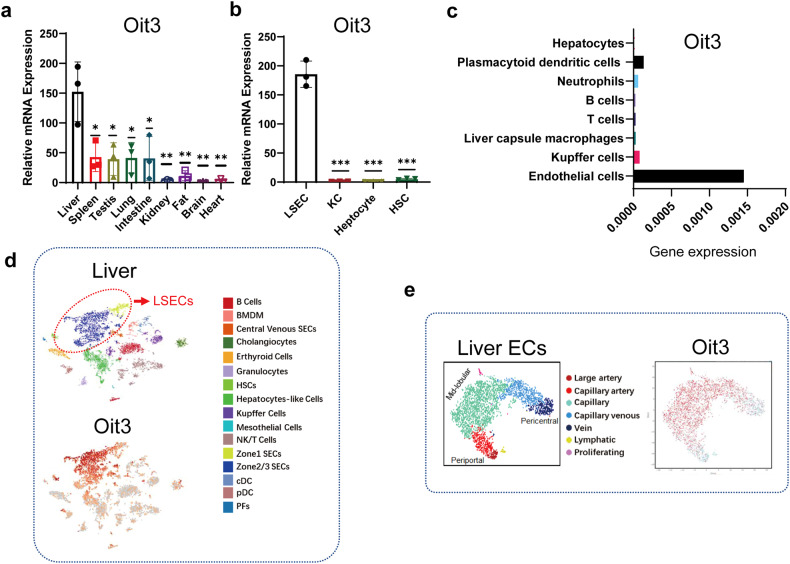
The LSEC-specific expression of Oit3. **a** The mRNA expression of Oit3 in liver, spleen, lung, brain, heart, testis, fat, intestine and kidney of mice, determined by qRT-PCR. β-actin was used as an internal control. **b** The mRNA expression of Oit3 in different kinds of liver cells, including hepatocytes, KCs, LSECs and HSCs. β-actin was used as an internal control. **c** The mRNA expression of Oit3 in hepatocytes, plasmacytoid dendritic cells, neutrophils, B cells, T cells, liver capsule macrophages, KCs and ECs, evaluated by the data of paired-cell sequencing of mice (GSE108561). **d** t-SNE plot revealed the spatial distribution of Oit3 in liver (GSE134037). **e** t-SNE plot revealed the spatial distribution of Oit3 in liver ECs (data from EC altas). Bars represent means ± SD, *n* = 3; *P < 0.05, **P < 0.01, ***P < 0.001

We then measured Oit3 and Dnase1l3 expression in distinct organs and different kinds of liver cells by RT-qPCR. Among distinct organs, the liver devoted a major contribution to Oit3 expression (Fig. 4a). Unlike Oit3, the expression level of Dnase1l3 in the spleen is comparable to that in the liver (Supplementary Fig. 1a). In the liver, striking mRNA levels of Oit3 and Dnase1l3 could be detected in LSECs, rather than KCs, hepatocytes, or HSCs (Fig. 4b, Supplementary Fig. 1b). The paired-cell sequencing of liver ECs also demonstrated that Oit3 and Dnase1l3 majorly originated from liver ECs (Fig. 4c, Supplementary Fig. 1c) (3). Besides, scRNA-seq data unveiled the identical spatial distribution of Oit3+ cells and LSECs (Fig. 4d). These sequencing data combined with the qRT-PCR analysis proved the liver EC origin of Oit3. Thereafter, western blot analyses confirmed that Oit3 was only expressed in the liver in various organs of mice (Supplementary Fig. 2a), and in the liver, it is mainly expressed in LSECs (Supplementary Fig. 2b).

### Oit3 particularly expressed in the midlobular liver ECs

**Fig. 5:**
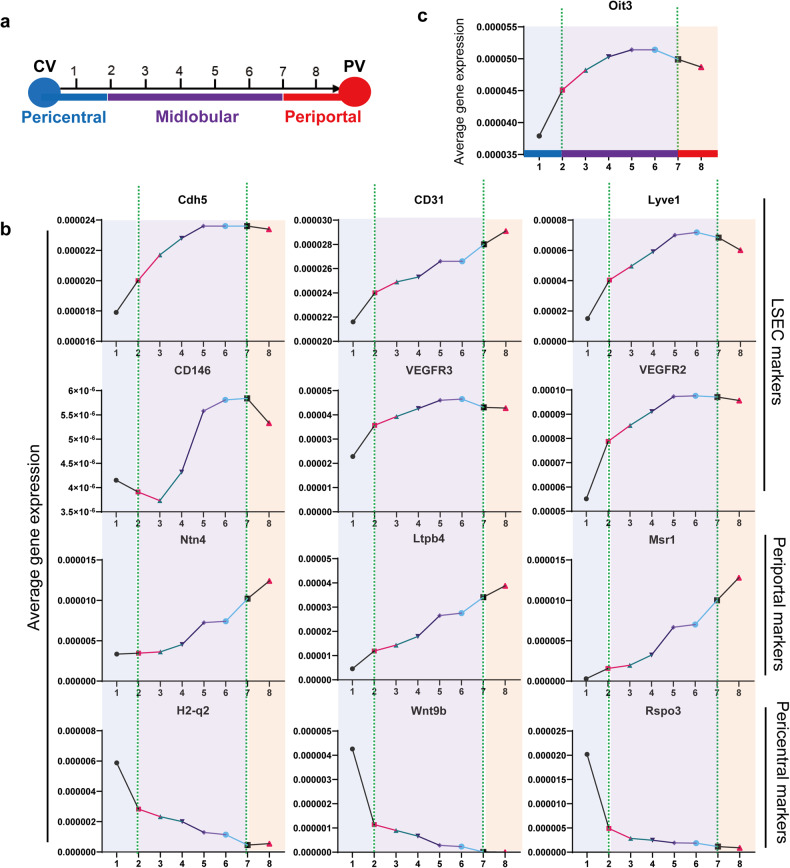
The spatial expression of endothelial markers. **a** A sketch mimicking the eight continuous regions from CV to PV. **b** The spatial expression of periportal, pericentral and EC marker genes in eight continuous regions (GSE108561). **c** The spatial expression of Oit3 in eight continuous regions of liver ECs (GSE108561)

Liver ECs can be roughly classified into three subpopulations, including pericentral, midlobular, and periportal ECs (Fig. 4e).^23,34,35^ According to the sequencing data collected from the EC atlas,^19^ Oit3 seemed to express dominantly in midlobular liver ECs, the majority of which were LSECs (Fig. 4e). To precisely illustrate the spatial gene expression of Oit3 in liver ECs, we delineated eight continuous regions from central vein (CV) to portal vein (PV) and estimated the spatial expression of several molecules in different regions of liver by paired-cell sequencing (Fig. 5a).^23^ We found that, from CV to PV, periportal marker genes like Ntn4, Ltpb4, and Msr1 gradually increased, which was opposite to the trend of pericentral marker genes such as H2-q2, Wnt9b, and Rspo3. Although the change of CD31 and CD146 seemed to be irregular, the spatial expression levels of other LSEC markers, including Cdh5, Lyve1, VEGFR2, and VEGFR3 were roughly the same (Fig. 5b). Next, Oit3 exhibited spatial expression identically compared to Lyve1 (Fig. 5c), suggesting Oit3, as well as Lyve1, represented the midlobular liver ECs, which constituted LSECs.

### OIT3 specifically expressed in human liver vascular endothelial cells

Taking into account the differences between humans and mice, we evaluated the expression patterns of Oit3 in humans. Similar to the expression pattern in mice, Oit3 is highly expressed in the liver in all organs of the human body (Supplementary Fig. 3a),^36^ and in the liver, it is only highly expressed in LSECs (Supplementary Fig. 3b).^35,37^

### Construction and validation of Oit3-CreERT2 reporter mice

**Fig. 6:**
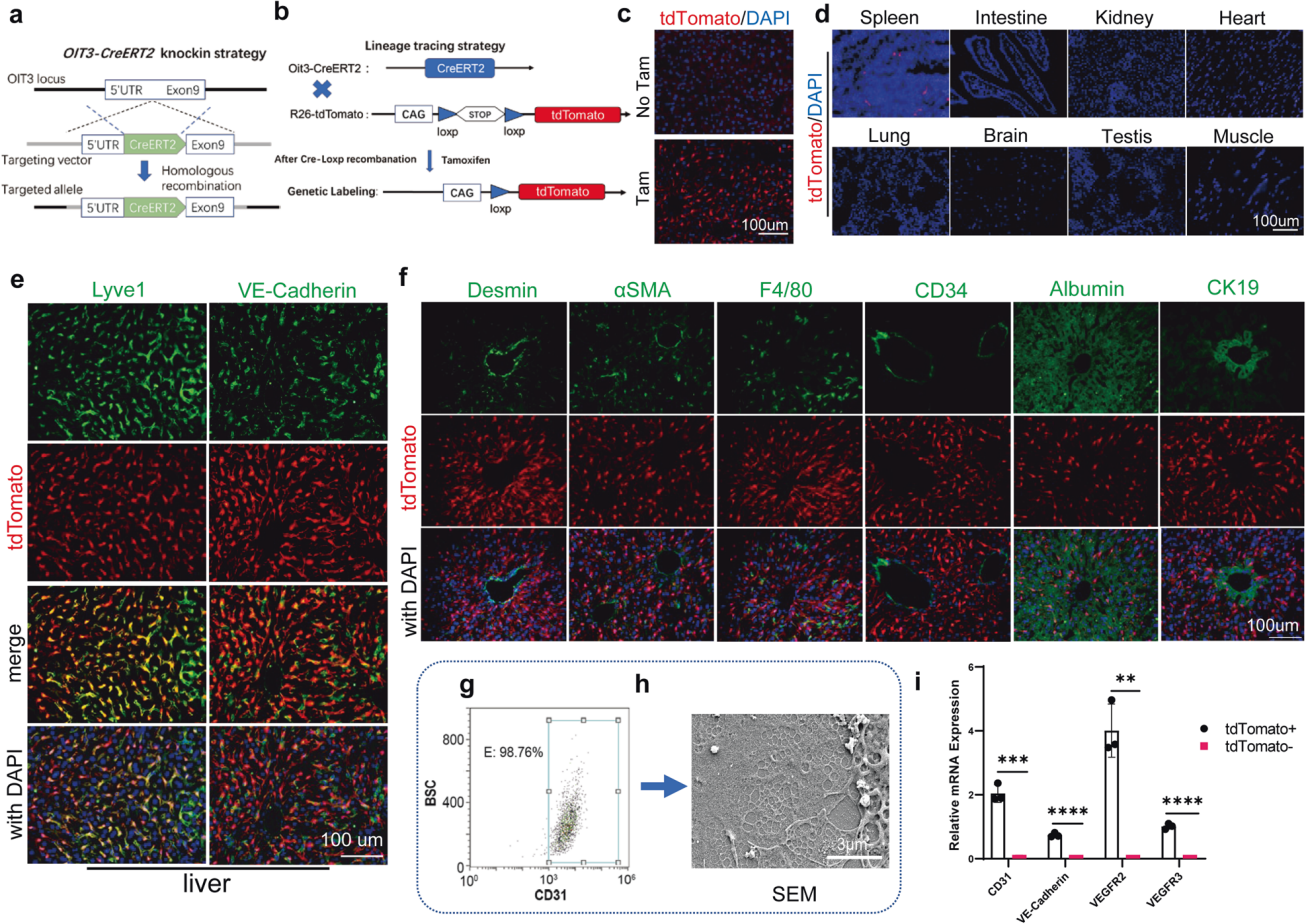
The generation of Oit3-CreERT2-tdTomato mice. **a** Strategy of the generation of Oit3-CreERT2 knockin allele. **b** Schematic image of the genetic lineage tracing strategy. **c** Microscopy of tdTomato in livers of Oit3-CreERT2-tdTomato mice with or without tamoxifen injection. **d** Microscopy of tdTomato and DAPI in spleen, lung, brain, kidney, testis, intestine, heart and muscle of Oit3-CreERT2-tdTomato mice treated with tamoxifen. **e** Immunofluorescent staining of VE Cadherin, Lyve1 and DAPI in livers of Oit3-CreERT2-tdTomato mice treated with tamoxifen. **f** Immunofluorescent staining of Desmin, αSMA, F4/80, CD34, Albumin, CK19 and DAPI in livers of Oit3-CreERT2-tdTomato mice treated with tamoxifen. **g** FACS analysis of CD31^+^ percentage in isolated tdTomato^+^ cells. **h** SEM showed the well-differentiated fenestrae of cultured tdTomato^+^ cells. **i** The comparison of mRNA expression of CD31, VE-Cadherin, VEGFR2 and VEGFR3 between tdTomato^+^ and tdTomato^-^ cells, determined by qRT-PCR. β-actin was used as an internal control. Bars represent means ± SD, *n* = 3; **P < 0.01, ***P < 0.001, ****P < 0.0001

Subsequently, we constructed inducible Oit3-driven CreERT2 transgenic mice. The strategy of the construction was displayed in Fig. 6a and the detailed technique was mentioned in the methods. We then crossed the established Oit3-CreERT2 knockin mice with Rosa26-tdTomato reporter mice to obtain Oit3-CreERT2-tdTomato mice (Fig. 6b), of which Oit3-driven cells could be labeled with tdTomato once the Cre recombinase was activated by tamoxifen.^38,39^

As expected, the liver of tamoxifen-treated Oit3-CreERT2-tdTomato mice exhibited significant red fluorescence under the investigation of the microscope (Fig. 6c). We also detected tdTomato in the spleen, lung, brain, kidney, testis, intestine, heart and muscle of Oit3-CreERT2-tdTomato mice. None of the above organs showed the appearance of tdTomato, except for the spleen, which displayed slight red fluorescence (Fig. 6d). Next, we clarified whether Oit3-driven tdTomato was labeled on LSECs. By immunofluorescent (IF) staining, we found that spontaneous tdTomato largely co-localized with Lyve1 and VE-Cadherin in the livers of Oit3-CreERT2-tdTomato mice, proving the successful label of LSECs by tdTomato (Fig. 6e). Besides, we distinguished tdTomato^+^ cells with other kinds of liver cells. IF staining confirmed that, tdTomato could not be overlaid with Desmin, αSMA, F4/80, CD34, Albumin, or CK19 (Fig. 6f), implying that Oit3 seldom expressed in HSCs, KCs, large artery ECs, hepatocytes, and cholangiocytes.

Ultimately, we evaluated Oit3^+^ LSECs ex vivo. tdTomato^+^ cells were collected from isolated liver nonparenchymal cells (NPCs) via fluorescence-activated cell sorting (FACS). Following flowcytometry analysis, we found that 98.76% of tdTomato^+^ cells were CD31 positive (Fig. 6g). Additionally, tdTomato^+^ cells, which were cultured in vitro and observed by scanning electronic microscope (SEM), exhibited well-differentiated LSEC-specific fenestrae (Fig. 6h). According to the qRT-PCR analysis, the mRNA expression of CD31, VE-Cadherin, VEGFR2, and VEGFR3 all enriched greatly in tdTomato^+^ cells (Fig. 6i). Collectively, these data proved that isolated Oit3^+^ cells were well-differentiated LSECs.

## Discussion

LSECs constituting the largest proportion of liver NPCs,^40^ regulate liver homeostasis and diseases through angiocrine factors. Well-differentiated LSECs inhibit HSC activation and nourish hepatocyte proliferation^41–43^ while dedifferentiated LSECs lose fenestrae and develop basement membrane, leading to HSC activation and hepatocyte damage.^1,3,17^ Thus, exploring the mechanisms involved in LSEC biology and pathology is of great importance.

Traditional scientific investigations have encountered challenges in identifying a distinctive biomarker for LSECs. This is due to the common embryonic origin shared by all endothelial cells found in various organs, which results in shared characteristics and functions.^4^ Despite these similarities, LSECs possess remarkable phenotypic and functional attributes that are influenced by their unique microenvironment within the liver. LSECs play a vital role as guardians, patrolling the bloodstream through the portal vein, which carries both beneficial nutrients and harmful toxins from the gastrointestinal tract.^21^ Working alongside Kupffer cells, LSECs form an exceptional scavenging system within the liver.^3,44^ Unlike other organ-specific endothelial cells, LSECs lack a basal membrane and exhibit heightened permeability. This exceptional adaptation allows LSECs to act as dynamic filters, carefully regulating the passage of various substances in and out of the liver.^3^ By utilizing advanced sequencing techniques, such as single-cell and pair-cell sequencing, we have successfully identified specific markers that are unique to LSECs. This breakthrough not only deepens our understanding of these specialized cells but also paves the way for further scientific exploration in elucidating cellular marker across different organs, leading to potential advances in therapeutic interventions.

Normally, LSECs are labeled by EC markers like Lyve1, CD31, VE-Cadherin, VEGFR2, or VEGFR3, and were isolated using anti-CD146 magnetic beads.^11^ According to the scRNA-seq data we provided in this manuscript, none of the above-mentioned genes is liver specific. Dnase1l3, which ranked No.1 of liver capillary EC marker genes in expression, is supposed to be a promising candidate. However, it exhibited the maximum expression in the spleen rather than the liver. Through multi-scRNA-seq analysis, we found that Oit3, one of the top 10 liver capillary EC marker genes, exhibits predominant hepatic expression and manifests selective localization in LSECs, endowing OIT3 with the status of an exemplary marker candidate. Interestingly, the microscopic observation on organs collected from Oit3-CreERT2-tdTomato mice showed that Oit3-driven tdTomato manifested slightly in the spleen. This phenomenon was corroborated by the sequencing data of the EC atlas. Therefore, we cannot conclude that Oit3 is expressed absolutely within the liver, but we should admit that Oit3 is up to now one of the best marker genes to distinguish LSECs. The Western blot validation of the single-cell sequencing analysis unveiled a fascinating revelation - the presence of a discrete yet discernible expression of the OIT3 protein within the hepatocytes and Kupffer cells. The presence of doublets and compromised cellular purity,^45^ often encountered during the intricate enzymatic digestion of hepatic tissue, provides an intriguing conjecture that may underlie the detection of OIT3 in non-LSEC cellular populations.

Our findings challenge the prevailing notion put forth by prior investigations, which posited that the predominant expression of OIT3 is concentrated in hepatocytes, rather than LSECs.^30,46^ However, we are inclined to infer that OIT3 predominantly manifests in LSECs, as earlier studies failed to acknowledge the intricate relationship between OIT3 and LSECs. This further exemplifies the advantageous potential of employing single-cell genomics for discerning cell-specific gene expression. Our study presents copious evidence substantiating the dominant expression of OIT3 primarily within LSECs. Nonetheless, there exists a pressing need for further exploration into its functional role within LSECs and its potential dynamics during pathological processes.

Liver endothelial cells, comparable to hepatocytes and hepatic stellate cells, demonstrate profound functional zonation patterns spanning the radial axis of the hepatic lobule.^47^ However, the precise repercussions of this remarkable spatial heterogeneity on liver pathology remain elusive. With the large improvement of scRNA-seq, the heterogeneity of ECs has been heatedly discussed in recent years. Liver ECs can be classified into three major populations in light of gene expression. Wnt2, Wnt9b, and Rspo3 were proven to be pericentral endothelial marker genes and Delta-like 4 (Dll4), EphrinB2, and Ltbp4 were reported to represent periportal liver ECs.^47^ Genes spatially expressed in the mid-lobule of liver ECs were poorly studied before. In this manuscript, utilizing the paired-cell sequencing data, we unveiled the midlobular distribution of Oit3. Thus, discriminating midlobular liver ECs by Oit3 seems to be rational in subsequent studies.

In this investigation, we primarily employed paired-cell sequencing to explore the hepatic localization of OIT3 RNA. With the advent of advanced spatial transcriptomics techniques, the zonation distribution of OIT3 will be subjected to further validation. It is worth highlighting that molecular expression patterns may undergo alterations under distinct pathological conditions.^47^ The data sources utilized in this study are constrained and may not comprehensively represent the specific genes associated with LSECs across all disease models. The zonation characteristics of OIT3 in liver pathologies, including steatosis, fibrosis, and hepatic regeneration, necessitate additional corroboration. Furthermore, the data employed in this investigation primarily stem from RNA analysis. As novel methodologies like single-cell proteomics come to the forefront, a more comprehensive exploration of the role of OIT3 in LSECs will ensue.^48^

To achieve LSEC-specific drug delivery, exosome or extracellular vesicle-loaded drug was modified to target Stab2 in previous findings.^49–51^ Considering the extrahepatic expression of Stab2, the therapeutic effect of this targeted delivery system was questionable. Once Oit3 was proven to be a hallmark gene of LSECs, targeting Oit3 provides us with a promising strategy to treat liver diseases with hepatic vasculature disorders. Besides, by virtue of the discovery of the LSEC origin of Oit3, we constructed Oit3-CreERT2 transgenic mice. This brand-new model may pave the way for the study of LSEC-associated liver diseases, such as sinusoidal obstructive syndrome (SOS). Moreover, with the growing interest in researching the crosstalk among distinct organs,^52–54^ the establishment of Oit3-CreERT2 transgenic mice offers a satisfactory model to elucidate the potential link between liver endothelium and cells of other remote organs. It can be seen that this mouse model will help us to better understand the complexity of LSECs in liver diseases and systemic disorders.

In this investigation, we present a pioneering revelation that Oit3 emerges as a formidable hallmark gene for LSECs. However, we must acknowledge that our examination predominantly relied upon scRNA-seq data obtained from murine models. The applicability of Oit3 in effectively delineating human LSECs amidst liver pathologies has regrettably remained unexplored and necessitates further deep-diving. Additionally, it is imperative to duly consider the subtle issue of tdTomato contamination in the spleen of the established reporter mice. Given the present spectrum of knowledge, the identification of an endothelial cell marker gene that exhibits pure, liver-specific expression appears to represent a herculean challenge. To facilitate the design of subsequent research utilizing this model, careful evaluation of the potential impact of spleen-derived ECs on the liver must be undertaken.

## Materials and Methods

### Mice

All mice in the study were kept in a pathogen-free facility. C57BL/6 and Oit3-CreERT2 mice were obtained from GemPharmatech (Jiangsu, China), while the R26-tdTomato mice were purchased from Jackson Laboratory (stock #007905, MGI:3809523). The Oit3-CreERT2 mouse line was generated by GemPharmatech using the CRISPR/Cas9 technology. In brief, P2A-CreERT2 was inserted into exon 9 of the Oit3 gene to create a knock-in line of Oit3-CreERT2 through homologous recombination. The Oit3-CreERT2 mouse was then crossed with the R26-tdTomato responsive reporter line, resulting in the Oit3-CreERT2-R26-tdTomato mouse line. Tamoxifen injections were used to induce Cre-loxP recombination and label Oit3+ cells. Polymerase chain reaction (PCR) analysis was performed on tail DNA to determine the genotypes of the mice. PCR primers were designed to target the correct allele (Oit3-CreERT2 primers: 5′-TGACTGTCCTTATCCCTTCAGGAG-3′; 5′-CATGTCCATCAGGTTCTTGCGAAC-3′). Male mice at six weeks of age were intraperitoneally injected with tamoxifen (100 mg/kg, Sigma, T5648) once daily for five injections. The mice were used for further experiments one week after the last injection.

### Isolation and culture of liver cells

Using a modified two-step collagenase perfusion method as previously described, hepatocytes, KCs, LSECs, and HSCs were isolated from mice. First, mice were anesthetized with 1% pentobarbital sodium and perfused with 25 ml of a prewarmed buffer containing no calcium or magnesium, but with EDTA (9 g/L NaCl, 0.416 g/L KCl, 2.1 g/L NaHCO3, 1.08 g/L glucose, 4.8 g/L Hepes, 0.58 g/L EDTA). This perfusion was performed for approximately 5 min, with the portal vein severed for drainage. Next, the liver was perfused with another 25 ml of a prewarmed buffer containing type IV collagenase and calcium, and magnesium (9 g/L NaCl, 0.416 g/L KCl, 2.1 g/L NaHCO3, 1.08 g/L glucose, 4.8 g/L Hepes, 0.222 g/L CaCl2, 0.4065 g/L MgCl2·6H2O, 0.4 mg/ml collagenase IV) for 5 min. After removing the liver, it was finely minced using gentle MACS C-tubes (Miltenyi Biotec, Bergisch Gladbach, Germany) and a tissue dissociator (Miltenyi) in 5 ml of digestive perfusion buffer containing DNase I (100 µg/ml, Roche, Basel, Switzerland). The minced liver was then incubated for 30 min at 37 °C with gentle shaking. The resulting solution was passed through a 100-µm cell mesh to obtain a single-cell suspension. Hepatocytes were removed through three rounds of centrifugation at 50 × *g* for 3 min. The remaining hepatic NPC were collected by centrifugation at 400 × *g* for 7 min. These cells were then resuspended in 4 ml of 17.6% OptiPrep (Axis-Shield, Oslo, Norway). Additionally, 4 ml of 11.5% OptiPrep and 2 ml of DMEM were layered sequentially on top of the suspension. After centrifugation at 1400 × *g* for 20 min without any breaks, HSCs were obtained from the interface between the top and intermediate layer. The KCs and LSECs fractions were obtained from the interface between the bottom and intermediate layer and were further purified using magnetic beads coated with CD146 and anti-F4/80 antibodies (Supplementary Table 1), following the manufacturer’s instructions.

### Flow cytometry

To prepare a single-cell suspension of NPCs, the following steps were followed. Cells were incubated with the respective antibodies (Supplementary Table 1) in fluorescence-activated cell sorter (FACS) buffer, which consisted of PBS (Gibco,10010-023) containing 1% BSA (AMRESO, E588-100G) and 0.01% sodium azide, for a duration of 30 min. After incubation, the samples were analyzed and sorted using a MA900 flow cytometer (SONY), and the resulting data were then analyzed using the MA900 software. Unstained NPCs were used for gate determination, and isotype antibodies were used as negative controls.

### Immunostaining and image acquisition

To prepare cryosections of organs for immunofluorescence analysis, the following steps were performed. Firstly, mice were perfused with PBS and the organ samples were fixed in 4% paraformaldehyde (PFA) for a duration of 2 h. Subsequently, the samples were washed with PBS, dehydrated overnight in graded sucrose solutions at 4 °C, and finally snap-frozen in optimal cutting temperature compound (TissueTek, Sakura) at −80 °C.

For microscopy, cryosections of the mouse liver (8 µm) were allowed to air-dry at room temperature for 2 h, followed by a wash with PBS. Afterward, the samples were blocked and permeabilized using QuickBlockTM blocking buffer (Beyotime, Haimen, China) for a period of 1 h at room temperature. Sections were then incubated with the primary antibodies (previously mentioned) at 4 °C overnight.

Once the sections were washed with PBS, they were incubated with the secondary fluorescent antibodies (as listed in Supplementary Table 1) at room temperature for a duration of 2 h. To visualize the nuclei, a counterstain of DAPI (Servicebio, Wuhan, China) was applied. The resulting images were captured using a fluorescence microscope (BX51, Olympus).

### SEM

To observe in vitro cultured tdTomato+ cells, the cells were first washed with PBS and then fixed using a 2.5% glutaraldehyde solution. The collected samples were subsequently dehydrated in ethanol, dried using a vacuum desiccator, mounted on aluminum stubs, sputter-coated with gold, and finally examined under an S-3400N scanning electron microscope (Hitachi, Tokyo, Japan).

### qRT-PCR

For the preparation of total RNA, TRIzol (Invitrogen) was used, followed by reverse transcription into cDNA using the PrimeScrip RT reagent kit (TaKaRa Biotechnology, Dalian, China). Quantitative real-time PCR (qPCR) was then conducted using the SYBR premix ExTaqII (TaKaRa Biotechnology) and the Applied Biosystems 7500 Realtime PCR system (Applied Biosystems, Foster City, CA). β-actin was utilized as a reference control (Supplementary Table 2).

### Western blotting

Protein extraction was performed using RIPA lysis buffer supplemented with 10 mM phenylmethanesulfonyl fluoride. The quantification of protein was carried out using the BCA protein quantitative kit (Thermo Fisher Scientific, Rockford, IL). The protein samples were subjected to SDS-PAGE gel electrophoresis for separation and transferred onto polyvinylidene fluoride membranes. Following this, the membranes were blocked with 5% skimmed milk powder and incubated overnight at 4 °C with primary antibodies. Subsequently, the membranes were washed with TBST and incubated with secondary HRP-conjugated antibodies for 2 h at room temperature, followed by another round of washing with TBST. Protein signals were detected using the ChemiDoc MP Imaging System (Bio-Rad, Hercules, CA, USA). The Supplementary Table S1 contains a list of the antibodies used in this study.

### Statistical analysis

The data were analyzed using GraphPad software (version 6.02) and presented as means ± standard deviation (SD). A two-tailed Student’s *t* test was employed to compare two groups, while differences among multiple groups were evaluated using one-way ANOVA followed by Bonferroni’s post hoc test. A significance level of *P* < 0.05 was used to determine statistical significance. The symbols *P < 0.05, **P < 0.01, ***P < 0.001, and ****P < 0.0001 denote different levels of significance, while “ns” indicates non-significance.

### Supplementary information

suppl data

## Data Availability

The datasets utilized in Fig. 1 and Fig. 4e can be accessed at: https://carmelietlab.sites.vib.be/en/softwaretools/scCycle (10.1016/j.cell.2020.01.015). The dataset featured in Fig. 2a and Fig. 3a is available through the National Center for Biotechnology Information (nih.gov) (10.1038/nature139920, 10.1074/mcp.M113.035600). The dataset employed in Fig. 2b-c can be found at: www.ebi.ac.uk/gxa/home. Figure 3’s dataset can be accessed at: (https://figshare.com/projects/Tabula_Muris_Transcriptomic_characterization_of_20_organs_and_tissues_from_Mus_musculus_at_single_cell_resolution/27733) (GSE109774) (10.1038/s41586-018-0590-4). Figure 4d’s dataset is available at GEO Accession Viewer (nih.gov) (GSE134037) (10.3389/fcell.2021.671081). The dataset used in Fig. 4c, Fig. 5, and Supplementary Fig. 1c can be accessed at GEO Accession Viewer (nih.gov) (GSE108561) (10.1038/nbt.4231). Finally, the dataset used in Supplementary Fig. 3b is available at: (http://bioinfo.life.hust.edu.cn/liverdb) (GSE185477, GSE124395) (10.1038/nbt.4231, 10.1002/hep4.1854). All data supporting the findings of this study are available from the corresponding author upon reasonable request.

## Code availability

The datasets utilized in Fig. 1 and Fig. 4e can be accessed at: https://carmelietlab.sites.vib.be/en/softwaretools/scCycle (10.1016/j.cell.2020.01.015). The dataset featured in Fig. 2a and Fig. 3a is available through the National Center for Biotechnology Information (nih.gov) (10.1038/nature139920, 10.1074/mcp.M113.035600). The dataset employed in Fig. 2b–c can be found at: www.ebi.ac.uk/gxa/home. Figure 3’s dataset can be accessed at: (https://figshare.com/projects/Tabula_Muris_Transcriptomic_characterization_of_20_organs_and_tissues_from_Mus_musculus_at_single_cell_resolution/27733) (GSE109774) (10.1038/s41586-018-0590-4). Figure 4d’s dataset is available at GEO Accession Viewer (nih.gov) (GSE134037) (10.3389/fcell.2021.671081). The dataset used in Fig. 4c, Fig. 5, and Supplementary Fig. 1c can be accessed at GEO Accession Viewer (nih.gov) (GSE108561) (10.1038/nbt.4231). Finally, the dataset used in Supplementary Fig. 3b is available at (http://bioinfo.life.hust.edu.cn/liverdb) (GSE185477, GSE124395) (10.1038/nbt.4231, 10.1002/hep4.1854).
