# Supplementary material for: Oit3, a promising hallmark gene for targeting liver sinusoidal endothelial cells
Source: Signal Transduct Target Ther. 2023 Sep 12;8:344. doi: 10.1038/s41392-023-01621-2 (PMC10495338; doi:10.1038/s41392-023-01621-2)
Supplement: Supplementary file 1 — suppl data [file 41392_2023_1621_MOESM1_ESM.docx]

**Supplementary Materials for**

**Oit3, a promising hallmark gene for targeting liver sinusoidal endothelial cells**

Zhi-Wen Li^1,#^, Bai Ruan^1,2,#^, Pei-Jun Yang^1,#^, Jing-Jing Liu^1^,

Ping Song^1^,Juan-Li Duan^1^,Lin Wang^1,*^

Correspondence to: Lin Wang (fierywang@163.com)

**This PDF file includes:**

Supplemental Methods

Supplementary Figure 1-3 with their legends

Supplementary Table 1-2

**Supplemental Methods:**

**Mice**

All mice in the study were kept in a pathogen-free facility. C57BL/6 and Oit3-CreERT2 mice were obtained from GemPharmatech (Jiangsu, China), while the R26-tdTomato mice were purchased from Jackson Laboratory (stock #007905, MGI:3809523). The Oit3-CreERT2 mouse line was generated by GemPharmatech using the CRISPR/Cas9 technology. In brief, P2A-CreERT2 was inserted into exon 9 of the Oit3 gene to create a knock-in line of Oit3-CreERT2 through homologous recombination. The Oit3-CreERT2 mouse was then crossed with the R26-tdTomato responsive reporter line, resulting in the Oit3-CreERT2-R26-tdTomato mouse line. Tamoxifen injections were used to induce Cre-loxP recombination and label Oit3+ cells. Polymerase chain reaction (PCR) analysis was performed on tail DNA to determine the genotypes of the mice. PCR primers were designed to target the correct allele (Oit3-CreERT2 primers: 5’-TGACTGTCCTTATCCCTTCAGGAG-3’; 5’-CATGTCCATCAGGTTCTTGCGAAC-3’). Male mice at six weeks of age were intraperitoneally injected with tamoxifen (100 mg/kg, Sigma, T5648) once daily for five injections. The mice were used for further experiments one week after the last injection.

**Isolation and Culture of Liver Cells**

Using a modified two-step collagenase perfusion method as previously described, hepatocytes, KCs, LSECs, and HSCs were isolated from mice. First, mice were anesthetized with 1% pentobarbital sodium and perfused with 25 ml of a prewarmed buffer containing no calcium or magnesium, but with EDTA (9 g/L NaCl, 0.416 g/L KCl, 2.1 g/L NaHCO3, 1.08 g/L glucose, 4.8 g/L Hepes, 0.58 g/L EDTA). This perfusion was performed for approximately 5 minutes, with the portal vein severed for drainage. Next, the liver was perfused with another 25 ml of a prewarmed buffer containing type IV collagenase and calcium, and magnesium (9 g/L NaCl, 0.416 g/L KCl, 2.1 g/L NaHCO3, 1.08 g/L glucose, 4.8 g/L Hepes, 0.222 g/L CaCl2, 0.4065 g/L MgCl2·6H2O, 0.4 mg/ml collagenase IV) for 5 minutes. After removing the liver, it was finely minced using gentle MACS C-tubes (Miltenyi Biotec, Bergisch Gladbach, Germany) and a tissue dissociator (Miltenyi) in 5 ml of digestive perfusion buffer containing DNase I (100 µg/ml, Roche, Basel, Switzerland). The minced liver was then incubated for 30 minutes at 37^◦^C with gentle shaking. The resulting solution was passed through a 100-µm cell mesh to obtain a single cell suspension. Hepatocytes were removed through three rounds of centrifugation at 50 × g for 3 minutes. The remaining hepatic NPC were collected by centrifugation at 400 × g for 7 minutes. These cells were then resuspended in 4 ml of 17.6% OptiPrep (Axis-Shield, Oslo, Norway). Additionally, 4 ml of 11.5% OptiPrep and 2 ml of DMEM were layered sequentially on top of the suspension. After centrifugation at 1,400 × g for 20 minutes without any breaks, HSCs were obtained from the interface between the top and intermediate layer. The KCs and LSECs fractions were obtained from the interface between the bottom and intermediate layer and were further purified using magnetic beads coated with CD146 and anti-F4/80 antibodies (Supplementary Table 1), following the manufacturer's instructions.

**Flow Cytometry**

To prepare a single cell suspension of NPCs, the following steps were followed. Cells were incubated with the respective antibodies (Supplementary Table 1) in fluorescence-activated cell sorter (FACS) buffer， which consisted of PBS (Gibco,10010-023) containing 1% BSA (AMRESO, E588-100G) and 0.01% sodium azide, for a duration of 30 minutes. After incubation, the samples were analyzed and sorted using a MA900 flow cytometer (SONY), and the resulting data were then analyzed using the MA900 software. Unstained NPCs were used for gate determination, and isotype antibodies were used as negative controls.

**Immunostaining and Image Acquisition**

To prepare cryosections of organs for immunofluorescence analysis, the following steps were performed. Firstly, mice were perfused with PBS and the organ samples were fixed in 4% paraformaldehyde (PFA) for a duration of 2 hours. Subsequently, the samples were washed with PBS, dehydrated overnight in graded sucrose solutions at 4°C, and finally snap-frozen in optimal cutting temperature compound (TissueTek, Sakura) at -80°C.

For microscopy, cryosections of the mouse liver (8 µm) were allowed to air-dry at room temperature for 2 hours, followed by a wash with PBS. Afterward, the samples were blocked and permeabilized using QuickBlockTM blocking buffer (Beyotime, Haimen, China) for a period of 1 hour at room temperature. Sections were then incubated with the primary antibodies (previously mentioned) at 4°C overnight.

Once the sections were washed with PBS, they were incubated with the secondary fluorescent antibodies (as listed in Supplementary Table 1) at room temperature for a duration of 2 hours. To visualize the nuclei, a counterstain of DAPI (Servicebio, Wuhan, China) was applied. The resulting images were captured using a fluorescence microscope (BX51, Olympus).

**SEM**

To observe in vitro cultured tdTomato+ cells, the cells were first washed with PBS and then fixed using a 2.5% glutaraldehyde solution. The collected samples were subsequently dehydrated in ethanol, dried using a vacuum desiccator, mounted on aluminum stubs, sputter-coated with gold, and finally examined under an S-3400N scanning electron microscope (Hitachi, Tokyo, Japan).

**qRT-PCR**

For the preparation of total RNA, TRIzol (Invitrogen) was used, followed by reverse transcription into cDNA using the PrimeScrip RT reagent kit (TaKaRa Biotechnology, Dalian, China). Quantitative real-time PCR (qPCR) was then conducted using the SYBR premix ExTaqII (TaKaRa Biotechnology) and the Applied Biosystems 7500 Realtime PCR system (Applied Biosystems, Foster City, CA). β-actin was utilized as a reference control (Supplementary Table 2).

**Western blotting**

Protein extraction was performed using RIPA lysis buffer supplemented with 10 mM phenylmethanesulfonyl fluoride. The quantification of protein was carried out using the BCA protein quantitative kit (Thermo Fisher Scientific, Rockford, IL). The protein samples were subjected to SDS-PAGE gel electrophoresis for separation and transferred onto polyvinylidene fluoride membranes. Following this, the membranes were blocked with 5% skimmed milk powder and incubated overnight at 4 °C with primary antibodies. Subsequently, the membranes were washed with TBST and incubated with secondary HRP-conjugated antibodies for 2 hours at room temperature, followed by another round of washing with TBST. Protein signals were detected using the ChemiDoc MP Imaging System (Bio-Rad, Hercules, CA, USA). The Supplementary Table S1 contains a list of the antibodies used in this study.

**Data and Code Availability**

The datasets utilized in Fig. 1 and Fig. 4e can be accessed at: <https://carmelietlab.sites.vib.be/en/softwaretools/scCycle> (doi:10.1016/j.cell.2020.01.015). The dataset featured in Fig. 2a and Fig. 3a is available through the National Center for Biotechnology Information ([nih.gov](http://nih.gov)) (doi:10.1038/nature139920, doi:10.1074/mcp.M113.035600). The dataset employed in Fig. 2b-c can be found at: [www.ebi.ac.uk/gxa/home](http://www.ebi.ac.uk/gxa/home). Fig. 3's dataset can be accessed at: (<https://figshare.com/projects/Tabula_Muris_Transcriptomic_characterization_of_20_organs_and_tissues_from_Mus_musculus_at_single_cell_resolution/27733>) (GSE109774) (doi:10.1038/s41586-018-0590-4). Fig. 4d's dataset is available at GEO Accession Viewer ([nih.gov](http://nih.gov)) (GSE134037) (doi: 10.3389/fcell.2021.671081). The dataset used in Fig. 4c, Fig. 5 and Supplementary Fig. 1c can be accessed at GEO Accession Viewer ([nih.gov](http://nih.gov)) (GSE108561) (doi:10.1038/nbt.4231). Finally, the dataset used in Supplementary Fig. 3b is available at: (<http://bioinfo.life.hust.edu.cn/liverdb>) (GSE185477, GSE124395) (doi:10.1038/nbt.4231, doi:10.1002/hep4.1854).

**Statistical analysis**

The data were analyzed using GraphPad software (version 6.02) and presented as means ± standard deviation (SD). A two-tailed Student’s t-test was employed to compare two groups, while differences among multiple groups were evaluated using one-way ANOVA followed by Bonferroni’s post hoc test. A significance level of P<0.05 was used to determine statistical significance. The symbols *P<0.05, **P<0.01, ***P<0.001, and ****P < 0.0001 denote different levels of significance, while "ns" indicates non-significance.


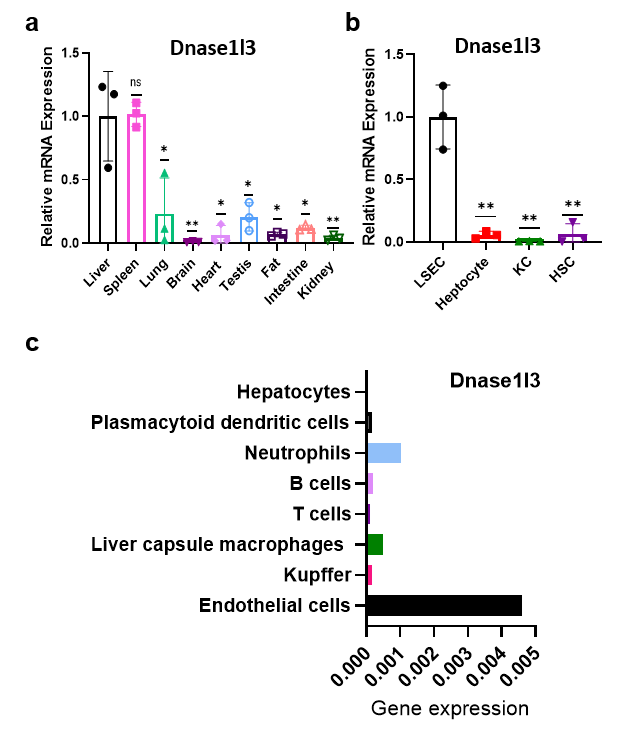


**Supplemental Figure 1: The gene expression of Dnase1l3.**

**a:** The mRNA expression of Dnase1l3 in liver, spleen, lung, brain, heart, testis, fat, intestine and kidney of mice, determined by qRT-PCR. β-actin was used as an internal control; **b:** The mRNA expression of Dnase1l3 in different kinds of liver cells, including hepatocytes, KCs, LSECs and HSCs. β-actin was used as an internal control; **c:** The mRNA expression of Dnase1l3 in hepatocytes, plasmacytoid dendritic cells, neutrophils, B cells, T cells, liver capsule macrophages, KCs and ECs, evaluated by the data of paired-cell sequencing of mice (GSE108561). Bars represent means ± SD, n=3; ns, no significance, *P < 0.05, **P < 0.01.


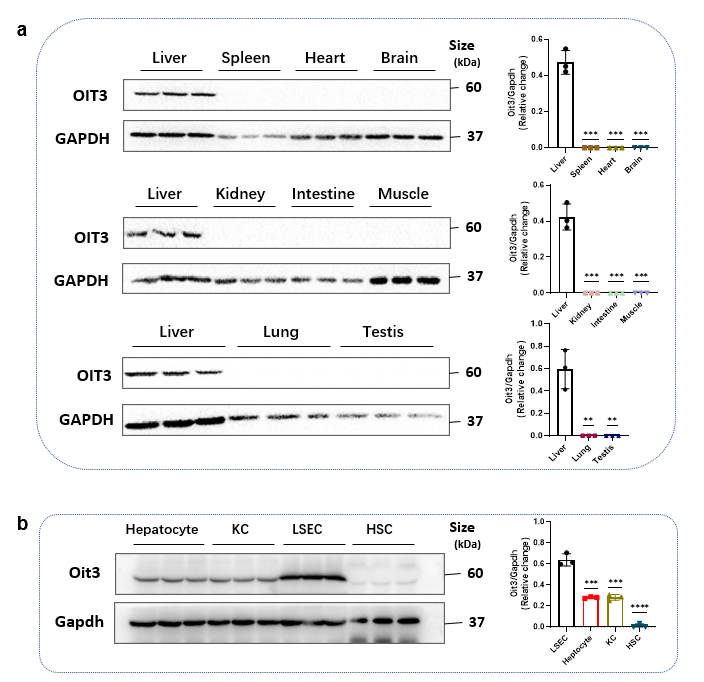


**Supplemental Figure 2: Western blot of Oit3 in** **mouse organs and liver cell types.**

**a:** Western blot of Oit3 in liver, spleen, heart, brain, intestine, kidney, muscle, lung and testis of mice, graph represents the relative percentage values of Oit3 (corrected for gapdh loading control); **b:** Western blot of Oit3 in different kinds of liver cells, including hepatocytes, KCs, LSECs and HSCs, graph represents the relative percentage values of Oit3 (corrected for gapdh loading control).


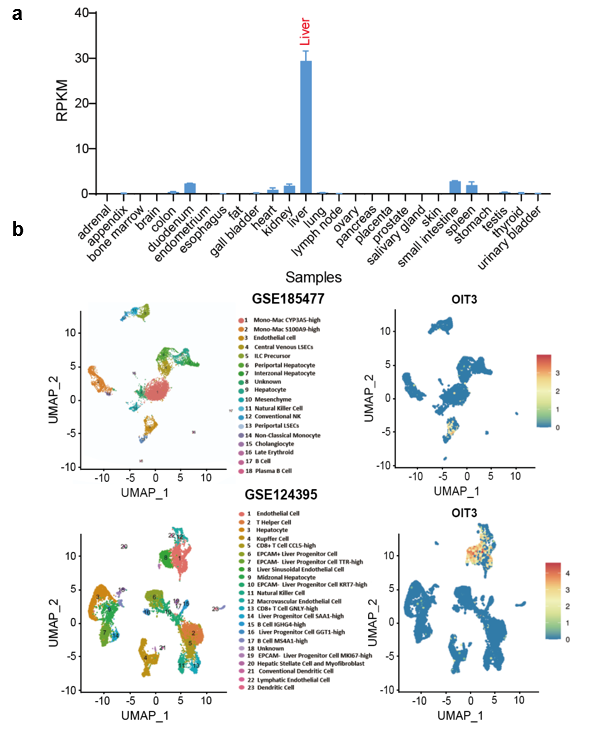


**Supplemental Figure 3: The gene expression of Oit3 in human livers.**

**a:** The relative expression of Oit3 in distinct organs of adults in human, data were collected from NCBI database; **b:** UMAP plot of Oit3 in human livers (GSE185477, GSE124395), data were collected from scLiverDB database.

**Supplementary Table 1**

| **Antibody** | **SOURCE** | **IDENTIFIER** |
| --- | --- | --- |
| Alexa Fluor® 488 anti-mouse CD31 (PECAM-1) Antibody | Biolegend | Cat #160207 |
| 7-AAD Viability Staining Solution | Biolegend | Cat #420403 |
| Anti-Desmin antibody [Y66] - Cytoskeleton Marker | Abcam | CAT #ab32362 |
| Rat Anti-Mouse Lyve-1 | Relia Tech | cat #103-M130 |
| Mouse VE-Cadherin Antibody | R&D Systems | Cat #AF1002 |
| Anti-alpha smooth muscle Actin antibody | Abcam | cat #ab124964 |
| F4/80 Monoclonal Antibody (BM8) | Invitrogen | Cat #14-4801-82 |
| Anti-CD34 antibody | Abcam | Cat #ab81289 |
| Donkey anti-Goat IgG (H+L) Cross-Adsorbed Secondary Antibody, Alexa Fluor™ 488 | Invitrogen | Cat #A-11055 |
| Donkey anti-Rat IgG (H+L) Highly Cross-Adsorbed Secondary Antibody, Alexa Fluor™ 488 | Invitrogen | Cat #A-21208 |
| Fluorescein (FITC) AffiniPure Goat Anti-Rabbit IgG (H+L) | Jackson Immuno Research | Cat #111-095-144 |
| Anti -Cytokeratin 19 Rabbit pAb | Servicebio | Cat#GB11197 |
| Anti-Mouse Serum Albumin Antibody | Abcam | Cat#ab19194 |
| Anti-LZP Antibody | Abcam | Cat#ab139190 |
| GAPDH Monoclonal Antibody | Proteintech | Cat#60004 |
| Goat Anti-Rabbit IgG H&L | Zenbio | Cat#550043 |
| Goat Anti-Mouse IgG H&L | Zenbio | Cat#550042 |

**Supplementary Table 2**

| **PCR** | | |
| --- | --- | --- |
| **primers** | **Forward, 5’-3’** | **Reverse, 5’-3’** |
| Oit3-CreERT2 mutant | TGACTGTCCTTATCCCTTCAGGAG | AAAGGTTGGCAGCTCTCATGTC |
| Oit3-CreERT2 wild type | TGACTGTCCTTATCCCTTCAGGAG | TCTTTGAGTCCAGAAGCTGAGGC |
| R26-tdTomato mutant | GGCATTAAAGCAGCGTATCC | CTGTTCCTGTACGGCATGG |
| R26-tdTomato wild type | AAGGGAGCTGCAGTGGAGTA | CCGAAAATCTGTGGGAAGTC |
| **qRT-PCR** | | |
| **primers** | **Forward, 5’-3’** | **Reverse, 5’-3’** |
| Oit3 | CACCTGCGGTCCTAGATCCT | GCAAAGAGGTTGATTCTGGGA |
| Dnase1l3 | CAATGGGCTTGGGACTCACT | TGGGTCGAAACTTCAGGGTG |
| CD31 | ACCGGGTGCTGTTCTATAAGG | TCACCTCGTACTCAATCGTGG |
| VEGFR2 | TTTGGCAAATACAACCCTTCAG | GCAGAAGATACTGTCACCACC |
| VEGFR3 | CTGGCAAATGGTTACTCCATGA | ACAACCCGTGTGTCTTCACTG |
| VE-Cadherin | CCACTGCTTTGGGAGCCTT | GGCAGGTAGCATGTTGGGG |
| β-actin | GGCTGTATTCCCCTCCATCG | CCAGTTGGTAACAATGCCATG |
